# All-Dielectric Color Filter with Ultra-Narrowed Linewidth

**DOI:** 10.3390/mi12030241

**Published:** 2021-02-27

**Authors:** Kai Xu, Yanlong Meng, Shufen Chen, Yi Li, Zhijun Wu, Shangzhong Jin

**Affiliations:** 1 College of Optical and Electronic Technology, China Jiliang University, Hangzhou 310018, China; xu_kai_194@aliyun.com (K.X.); yli@cjlu.edu.cn (Y.L.); jinsz@cjlu.edu.cn (S.J.); 2 Key Laboratory for Organic Electronics and Information Displays and Jiangsu Key Laboratory for Biosensors, Institute of Advanced Materials (IAM), Jiangsu National Synergetic Innovation Center for Advanced Materials (SICAM), Nanjing University of Posts and Telecommunications (NUPT), 9 Wenyuan Road, Nanjing 210023, China; iamsfchen@njupt.edu.cn; 3 Fujian Key Laboratory of Light Propagation and Transformation, College of Information Science and Engineering, Huaqiao University, Xiamen 361021, China; zhijunwu@hqu.edu.cn

**Keywords:** color filter, ultra-narrow linewidth, distributed Bragg reflection (DBR)

## Abstract

In this paper, a transmissive color filter with an ultra-narrow full width at half of the maximum is proposed. Exploiting a material with a high index of refraction and an extremely low extinction coefficient in the visible range allows the quality factor of the filter to be improved. Three groups of GaP/SiO_2_ pairs are used to form a Distributed Brag reflector in a symmetrical Fabry-Pérot cavity. A band-pass filter which is composed of ZnS/SiO_2_ pairs is also introduced to further promote the purity of the transmissive spectrum. The investigation manifests that a series of tuned spectrum with an ultra-narrow full width at half of the maximum in the full visible range can be obtained by adjusting the thickness of the SiO_2_ interlayer. The full width at half of the maximum of the transmissive spectrum can reach 2.35 nm. Simultaneously, the transmissive efficiency in the full visible range can keep as high as 0.75. Our research provides a feasible and cost-effective way for realizing filters with ultra-narrowed linewidth.

## 1. Introduction

Color filter is a kind of optical device which plays a significant role in many industrial fields, such as spectroscopy instruments, imaging sensors, and displays [1,2,3,4]. The precision and sensibility of many optical systems used in those fields are mainly decided by such an optical device. Generally, there are two types of optical filters: reflective filter and transmissive filter [5]. Usually, the reflective filter is realized by a perfect absorber, of which the filtered wavelengths are fully absorbed. As a result, its function is embodied in the reflective characteristic. On the contrary, the filtering function of a transmissive filter is obtained by permitting the required wavelengths to pass through. In comparison with the reflective filter, the transmissive filter is proper to be used in displays, charge-coupled devices, and hyperspectral imaging. With respect to the transmissive filter, the quality factor or the full width at half of the maximum (FWHM) of the transmissive spectrum is a principal characteristic if we want a filter with high accuracy and resolution [3]. In addition, the efficiency of the filter is also an important characteristic that should be considered. That is because a higher transmittance means a better energy use of incident light, which will be beneficial to reduce the dissipation of power and obtain a high intensity of the signal in optical systems using these filters.

Up to now, there have been many reports on filters realized by surface plasmons [6,7,8,9,10,11], Fabry–Pérot (FP) microcavities [12,13,14,15,16,17,18,19,20,21], grating guided-mode resonance [22,23,24,25,26,27,28], and Mie scattering [29,30,31,32,33,34,35]. However, high quality-factor filters realized by surface plasmons or Mie scattering always involve in many sophisticated nanostructures, such as nanohole array metal films [10,36], metal grating [23,37], and nanoparticle arrays. In comparison, the FP cavity based on multilayered thin films presents many advantages, such as easy to be fabricated, facilitating large-scale production. In recent years, there have been a large number of reports on the design of filters based on the FP cavity [16,20,21,38,39,40,41]. Using a dielectric layer sandwiched by two metallic layers to form an FP cavity is a convenient way, which is also named as metal-dielectric-metal (MDM) structure. However, the strong absorption of metallic layers limits the quality factor [39]. To further improve the quality factor of such structure, a distributed Bragg reflection (DBR) is a preferable candidate to substitute the metallic reflective mirror. Since the DBR structure is fabricated by various dielectric layers, the absorption in metallic layers can be avoided. So far, there are still few reports on filters with extremely narrow FWHM (<10 nm) in the visible range with a relatively simple DBR structure. 

In this paper, we propose a transmission filter structure based on an FP cavity. The reflective mirror is composed of GaP/SiO_2_ etalon. As a direct bandgap semiconductor material with a bandgap of 2.26 eV, GaP has a high refractive index in the visible range and can also be prepared by low-temperature atomic layer deposition technology or chemical vapor deposition technology, which is compatible with other thin-film deposition processing [42]. In addition, the absorption coefficient is close to zero when the wavelength is longer than 470 nm. A multilayer composed of ZnS/SiO_2_ pairs is used as a band-pass filter to improve the quality of the spectrum. Since the materials used have relatively small absorption in the visible range, it is expected to obtain a smaller FWHM through structural design. This research not only contributes to the preparation of large-area, high-resolution transmissive filters but also helps expand the application range of high-refractive-index semiconductor materials in the optical field.

## 2. Simulation Models

**Figure 1:**
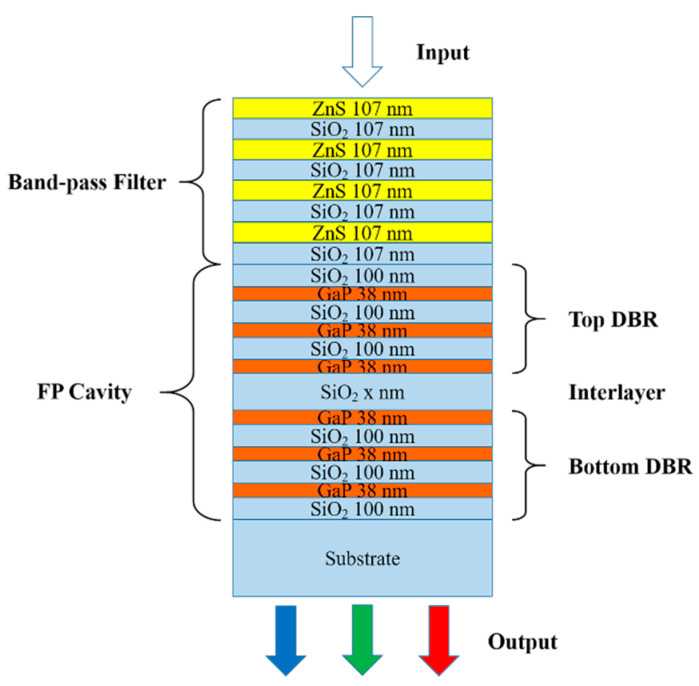
Structural diagram of the device used in simulation.

**Figure 2:**
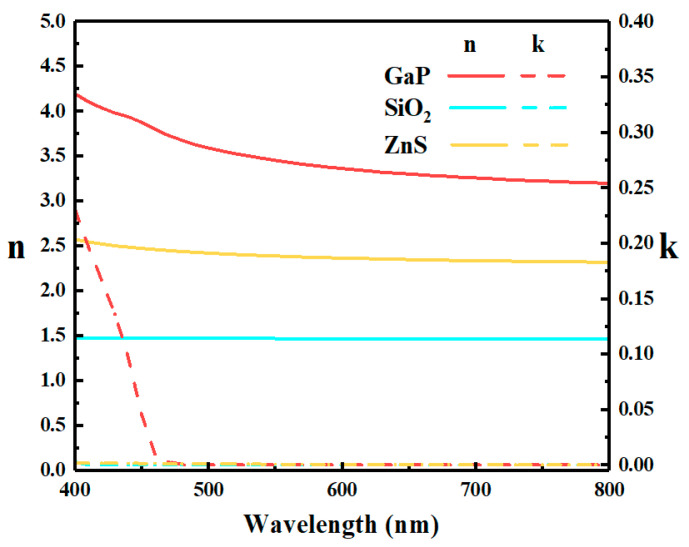
Refractive indices and extinction coefficients of materials used in simulation.

The simulated structure in this paper is shown in Figure 1. The light incidents from the top of the device and outputs from a glass substrate. As shown in Figure 1, the device’s structure can be divided into two components—a symmetrical FP cavity and a band-pass filter. The FP cavity is realized by two DBRs and a SiO_2_ interlayer. The DBR consists of several groups of GaP/SiO_2_ pairs. The thicknesses of GaP and SiO_2_ in each group and the number of groups have been optimized for obtaining a transmissive spectrum with an ultra-narrow FWHM and high intensity. The ZnS/SiO_2_ multilayer is set on top of the cavity as a band-pass filter for the purpose of improving the purity of the transmissive spectrum. The refractive indices and extinction coefficients of various materials used in the simulation are shown in Figure 2. All the data are quoted from other reports [43,44,45].

A finite-difference time-domain (FDTD) simulation was performed by using commercial software (Version 8.24, Ansys Lumerical, Inc, Vancouver, BC, Canada). In the simulation, a plane electromagnetic (EM) wave was used as an excitation source. The intensity of the electric field was set to 1, and the incident direction was the z backward. The boundary condition in the z-direction and other directions were perfect match layer (PML) and periodic boundary conditions, respectively. In order to simulate the performance of the device in different polarization states, two polarization, i.e., transverse magnetic (TM), transverse electric (TE) modes, were used. In TM and TE modes, the polarization directions of the electric field were set to parallel to and perpendicular to the plane of incidence. A Broadband Fixed Angle Source Technique (BFAST) was used to simulate the transmissive spectra of the device as the incidence of the wide-band EM wave is oblique. A monitor of electric field power was located on the opposite side of the device to test the transmittance. In addition, a 2D electric field monitor was placed parallel to the incident plane to test the distribution of the electric field in each layer.

## 3. Results and Discussion

### 3.1. Optimization of the FP Cavity

As a principal component of the device, the FP cavity without a band-pass filter was optimized firstly. The main purpose of the optimization is to generate a kind of transmissive spectrum, which possesses an ultra-narrow FWHM and high transmittance simultaneously. It is well known that the spectral FWHM is determined by the reflectivity of the reflective ends, the refractive index, and the thickness of the interlayer [46]. Its expression is shown as follows:

(1) $$\mathrm{FWHM}=\frac{-c\ln(R_{1}R_{2})}{4\pi nd}$$

where *R*_1_ and *R*_2_ are the reflectivities of the reflective ends, *c* is the velocity of electromagnetic waves in vacuum, *n* is the refractive index of the interlay, and *d* is the physical thickness of the SiO_2_ interlayer. According to the equation, it is clear that the FWHM can be narrowed by increasing the reflectivities of the two DBRs in the proposed structure, the refractive index, and the thickness of the intermediate dielectric layer. Accordingly, the thicknesses of each layer and the number of GaP/SiO_2_ groups in the DBR will be the critical parameters, which should be optimized firstly. 

**Figure 3:**
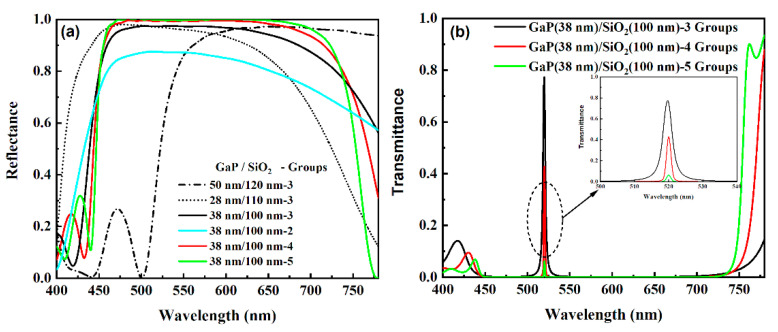
Reflective spectra (**a**) of the distributed Bragg reflection (DBR) reflectors with various structural parameters and transmissive spectra (**b**) of the Fabry–Pérot (FP) cavity with different numbers of GaP/SiO_2_ pairs. The inset of the left chart is an enlargement of the transmissive spectra at the center wavelengths.

In the beginning, the number of GaP/SiO_2_ groups is set to three. The thickness of each layer in the DBR is optimized by the Particle Swarm optimization method [47,48], which has been a mature module of the software. The aim of optimization is that the mean reflectance at the interface between the SiO_2_ interlayer and the DBR reaches the highest value. According to the result of optimization, the thicknesses of GaP and SiO_2_ in a single etalon are fixed to 38 nm and 100 nm, respectively. In order to illustrate the result of optimization clearly, the reflective spectra of the DBRs with different thicknesses of GaP and SiO_2_ are plotted in Figure 3. It can be seen from Figure 3a that the DBR with 38 nm-thick GaP and 100 nm-thick SiO_2_ shows a better reflection in the range from 450 nm to 750 nm, which covers the visible range exactly, than other DBRs. The reflectance of the optimized DBR reflector is higher than 0.8 in the full range. Especially in the range from 460 nm to 680 nm, the mean reflectance surpasses 0.9. Once the thicknesses of GaP and SiO_2_ are fixed, the number of GaP/SiO_2_ pairs can be determined facilely. It is obvious in Figure 3a that the reflectance of the DBR reflector becomes higher and higher, even close to 1.0 in the range from 450 nm to 750 nm, as the number of Gap/SiO_2_ pairs increases. According to such a result, it seems that the more numbers of Gap/SiO_2_ pairs the DBR has, the better performance the FP cavity possesses. However, the mean reflectance of the DBR is not the only factor that should be considered. Subsequently, the transmissive spectrum of the FP cavity with 170 nm-thick SiO_2_ interlayer is simulated and plotted in Figure 3b. It is straightforward that the intensity of the transmissive spectrum at the center wavelength, i.e., the resonant wavelength of the FP cavity, decreases dramatically as the number of groups increases. As shown in the inset in Figure 3b, the transmission at 520 nm almost vanishes when the number of GaP/SiO_2_ pairs reaches five. In another aspect, the more GaP/SiO_2_ pairs will lead to a more complicated fabrication processing and higher cost undoubtedly. Accordingly, three groups of GaP/SiO_2_ in the DBR are the best choice for the device. 

**Figure 4:**
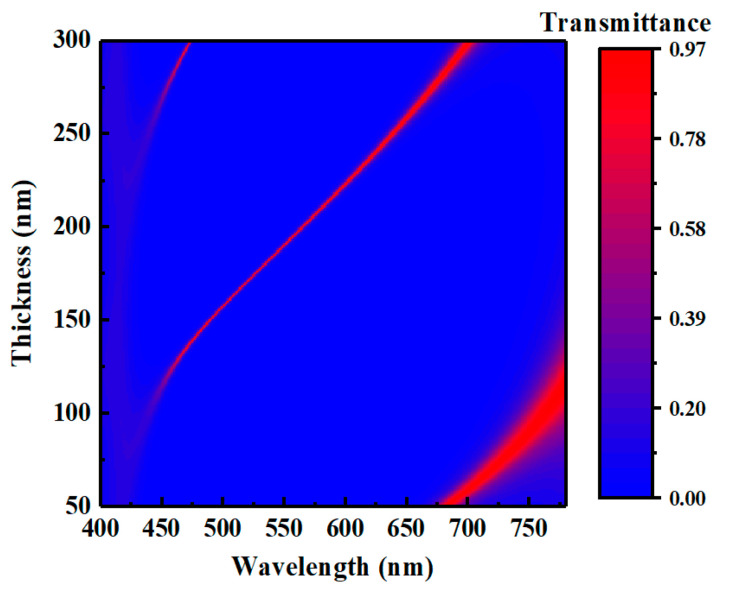
Evolution of the transmissive spectrum of the FP cavity as the thickness of interlayer changes.

Once the structural parameters of the DBR are fixed, the resonant wavelength of the FP cavity is determined only by the physical thickness of the SiO_2_ interlayer. The resonant wavelength of the FP cavity can be expressed as follows [46]:

(2) $$m\lambda_{m}=2dn(\lambda )$$

where d is the thickness of SiO_2_, n(λ) is the refractive index of SiO_2_, m is the eigenmode order, and $\lambda_{m}$ stands for the resonant wavelength of mth eigenmode. Figure 4 shows the evolution of the transmissive spectrum as the thickness of the intermediate dielectric layer SiO_2_ changes. It is obvious that three ultra-narrow bright bands appear in sequence, which represent three different orders of resonance mode, as the thickness of SiO_2_ increases from 50 nm to 300 nm. The peak of the spectrum at the resonant wavelength presents a redshift ranging from 420 nm to 700 nm as the thickness of SiO_2_ increases. That means three primary colors for displays can be obtained easily via such a structure. 

According to Equation (1), it can be known that the resonant wavelength of the eigenmode in a high order will present a narrower FWHM than that in low order when the interlayer of the FP cavity is fixed to a certain thickness. However, the high order indicates that the photons will travel in multiple rounds in the cavity before exiting from the other side. That will lead to a decay of the transmissive spectrum once loss exists in the dielectric layers [46]. As a result, using the resonant wavelength of an eigenmode in a higher-order to pursuit a narrower FWHM is not a suitable scheme for realizing a filter with high efficiency. In this paper, we choose the 1st order eigenmode to realize the function of the FP cavity.

**Figure 5:**
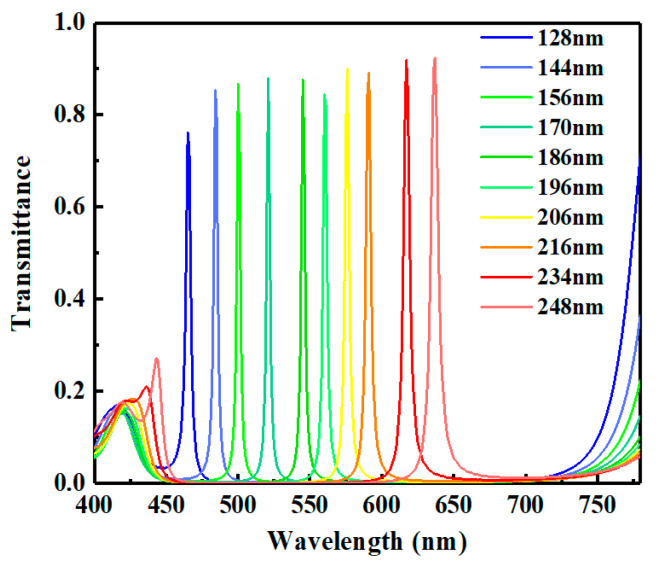
Transmissive spectra of FP cavity with different thicknesses of interlayer.

In order to analyze the characteristics of the FP cavity further, we extracted the transmissive spectra of the cavity with various thicknesses of the SiO_2_ interlayer (see Figure 5). It is straightforward that the center wavelength shifts from blue range to red range sequentially along with the increment of the thickness of SiO_2_. When the thickness increases from 128 nm to 248 nm, the center wavelength shifts from 465 nm to 636 nm. The variation range of the transmissive spectrum almost covers the visible range. The detailed data of the transmissive spectra are listed in Table 1. As shown in the table, the FWHMs of all spectra are less than 10 nm. In particular, the FWHMs of spectra corresponding to the center wavelengths at 465 nm, 520 nm, and 620 nm reach 6.46 nm, 2.05 nm, and 5.35 nm, respectively. Meanwhile, the transmittances can reach 0.756, 0.822, and 0.902, respectively. 

### 3.2. Optimization of the Band-Pass Filter

To improve the quality of the spectrum further, we introduce a band-pass filter on top of the FP cavity to stop the light, of which the wavelength is right consistent with the superfluous resonant wavelength, pass through. The structural parameters of the band-pass filter, which is composed of ZnS/SiO_2_ pairs, are optimized by utilizing a similar way to that used in the optimization of the FP cavity. 

**Figure 6:**
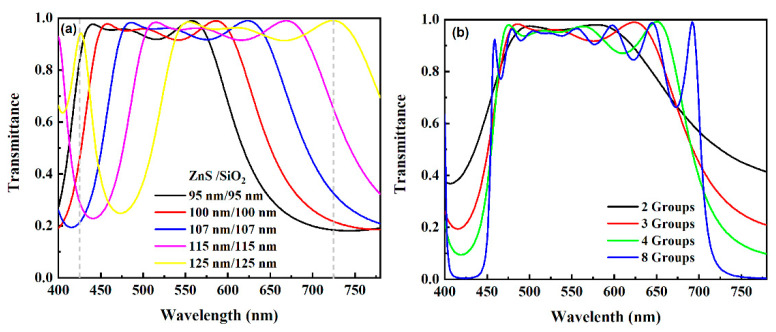
Transmissive spectra of the band-pass filters with different thicknesses (**a**) and numbers (**b**) of ZnS/SiO_2_ pairs. In the left chart, the number of ZnS/SiO_2_ is fixed to three. In the right chart, both of the thicknesses of ZnS and SiO_2_ are 107 nm.

The transmissive spectra of ZnS/SiO_2_ pairs with different thicknesses and number of pairs are compared in Figure 6. The main purpose for the optimization is depressing the spectral transmittance at the two ends of the visible range but allowing high spectral transmittance in the variation range of the resonant wavelength mentioned in Section 3.1. As shown in Figure 6a, the permitted band of the spectrum shifts to red as the thicknesses of ZnS and SiO_2_ increase. In comparison with other spectra, the spectrum of the band-pass filter with 107 nm-thick ZnS and 107 nm-thick SiO_2_ presents a permitted band that can cover the range from 465 nm to 636 nm. Though increasing the number of ZnS/SiO_2_ pairs is helpful for widening the bandwidth and forming a steep edge (see Figure 6b), the performance of band-pass filter with four groups of ZnS/SiO_2_ is sufficient to achieve the aim of optimization. In order to illustrate the results of optimization, the transmissive spectra of an integrated device are simulated. 

**Figure 7:**
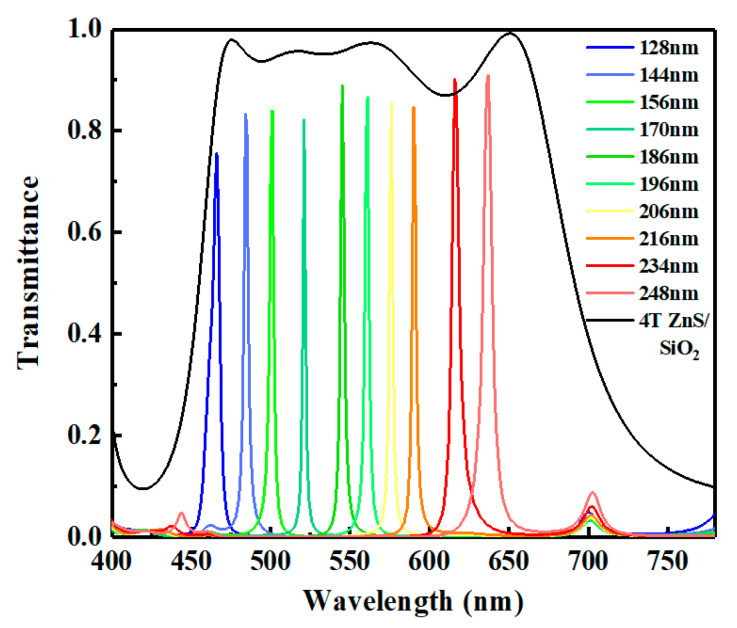
Transmissive spectra of integrated device with different thicknesses of SiO_2_ interlayer. Black solid line represents the transmittance curve of the chosen band-pass filter.

Figure 7 shows the transmissive spectra of the integrated device. It can be seen from the figure that the integrated device obtains an outstanding unimodal spectrum in the full visible range due to the suppression in the short wavelength range and long-wavelength range. In addition, the integrated device keeps excellent performance. As a result, the FWHMs of the transmissive spectra in the visible range are still less than 10 nm. The FWHMs at center wavelengths of 465 nm, 520 nm, and 620 nm are 6.70 nm, 2.35 nm, and 6.91 nm, respectively. Although the introduction of ZnS/SiO_2_ will weaken the transmittance slightly, the transmittance of the integrated device is still higher than 0.75 at various wavelengths. The transmittances reach 0.75, 0.82, and 0.90 at 465 nm, 520 nm, and 620 nm, respectively.

### 3.3. Evaluation of the Device’s Performance

In order to evaluate the chromaticity of the transmissive spectrum, the color coordinate of the transmissive spectrum in the 1931 Commission Internationale de L’Eclairage (CIE) coordinate system is calculated according to the CIE-XYZ tristimulus value, which is obtained from the following expressions:

(3) $$X=k\int_{}^{}R(\lambda )\bar{x}(\lambda )d\lambda$$

(4) $$Y=k\int_{}^{}R(\lambda )\bar{y}(\lambda )d\lambda$$

(5) $$Z=k\int_{}^{}R(\lambda )\bar{z}(\lambda )d\lambda$$

where k is the adjustment factor, $\bar{x}$, $\bar{y}$ and $\bar{z}$ are the optical efficiency functions, and R(λ) is the calculated spectrum. The coordinate can be calculated as follows:

(6) $$x=\frac{X}{X+Y+Z}$$

(7) $$y=\frac{Y}{X+Y+Z}$$

**Figure 8:**
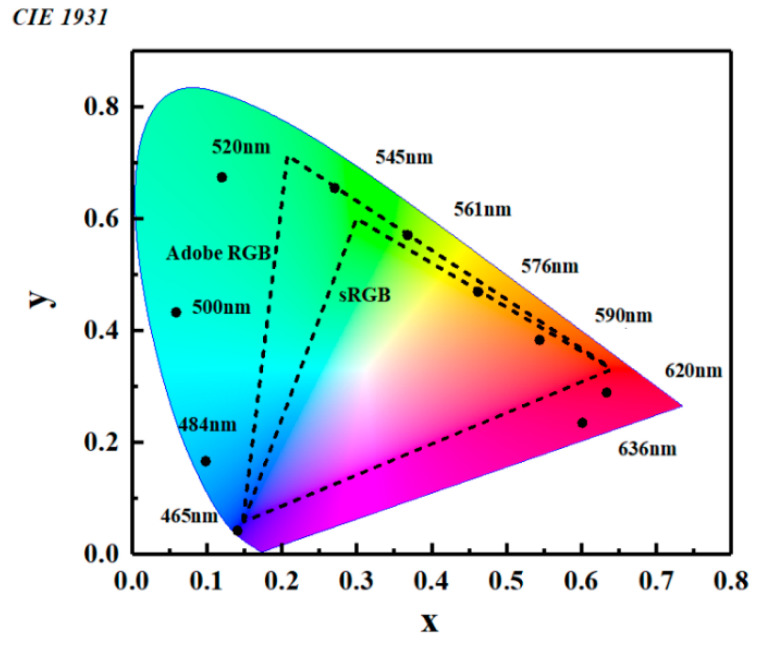
The chromaticities of transmissive spectra at different wavelengths. The two triangles stand for the gamut scale of Adobe RGB and sRGB.

The coordinates of spectra with different center wavelengths are shown in Figure 8. It can be seen from the figure that the chromaticities of various transmissive spectra are very close to the boundary of the CIE chart, especially for the spectrum with a center wavelength of 465 nm. It indicates that the spectrum has a high color purity. As the thickness of the intermediate layer increases, the corresponding coordinate varies from the region of blue to the regions of green, yellow, and red successively. As shown in Figure 8, most of the coordinates locate outside the Adobe RGB and sRGB chromatic gamut. That means a larger scale of chromatic gamut can be obtained, and more vivid colors can be reproduced via such filter.

**Figure 9:**
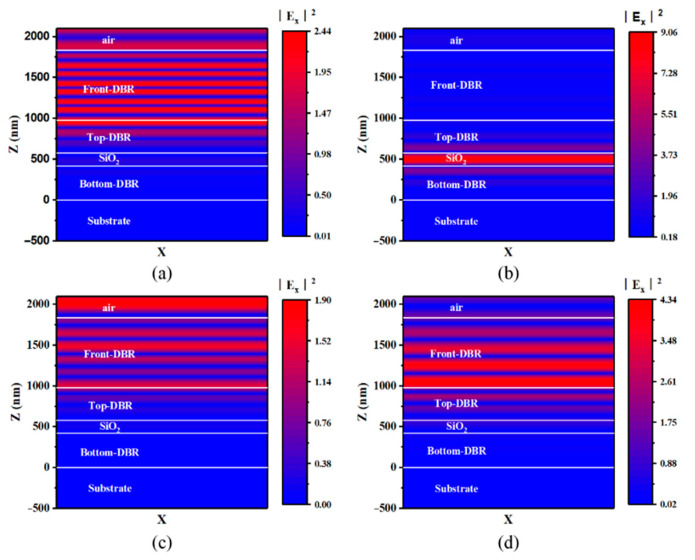
Distribution of electric field in filter at 460 nm (**a**), 520 nm (**b**), 600 nm (**c**), and 700 nm (**d**). The thickness of SiO_2_ interlayer in FP cavity is 170 nm.

Figure 9 shows the distribution of electric fields in each layer in the integrated device at various wavelengths when the thickness of SiO_2_ is 170 nm. Correspondingly, the center wavelength of the transmissive spectrum is 520 nm. The intensity of the electric field at 520 nm in the intermediate medium gets to the maximum in comparison with the electric fields at other wavelengths, such as 460 nm, 600 nm, and 700 nm. The consistency between the wavelength where the electric field in cavity reaches the maximum and the center wavelength of the transmissive spectrum indicates that the FP microcavity plays a decisive role in the filter device.

**Figure 10:**
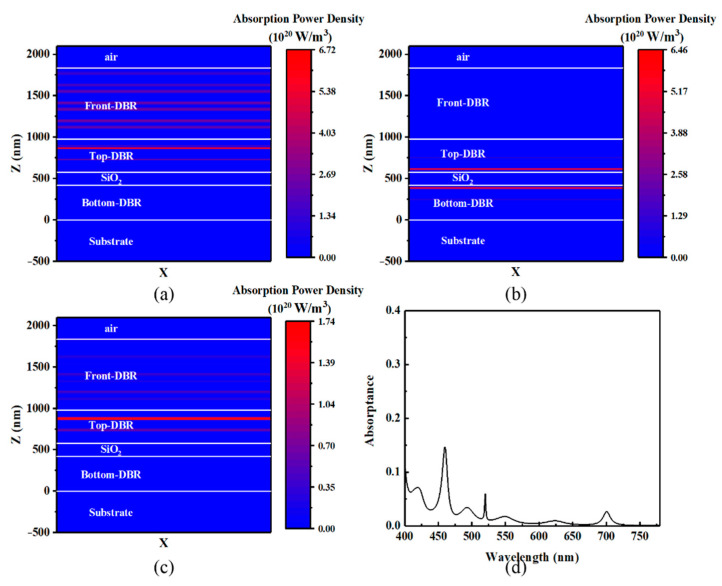
Distribution of absorption power density at 460 nm (**a**), 520 nm (**b**), and 700 nm (**c**) with a 170 nm-thick SiO_2_. Figure (**d**) presents the total absorption of the optical filter.

According to Equation (1), the ultra-narrow linewidth of the filter is ascribable to the high reflection on the GaP/SiO_2_ DBR, while the high transmissive efficiency is attributable to the low absorption of the device. Figure 10b shows the absorbed power density in each layer at various wavelengths. It can be seen in Figure 10a that a slight absorption occurs in the GaP layer, which is adjacent to the intermediate layer. Accompany with the diminishing of reflectivity at the resonant wavelength, the transmissive efficiency at resonant wavelength gets to a very high value according to the formula A(λ) + T(λ) + R(λ) = 1. Figure 10d shows an overall absorption of the device in the full visible range. It can be seen from the figure that absorption in the full range is very low except for a strong peak near 460 nm and a small absorption peak near 700 nm. As shown in Figure 10a,c, the strong absorptions at these wavelengths occur in the top DBR of the FP cavity and the band-pass filter. That is because the GaP and SiO_2_ layers in the DBR forms some submicrocavities as well as the ZnS and SiO_2_ layers in the band-pass filter. When the wavelengths of incident light satisfy the resonant conditions in those submicrocavities, there will be an enhancement of the electric field in the interlayer. Thus, some discrete absorbing peaks at those resonant wavelengths will exist in the absorption curve.

**Figure 11:**
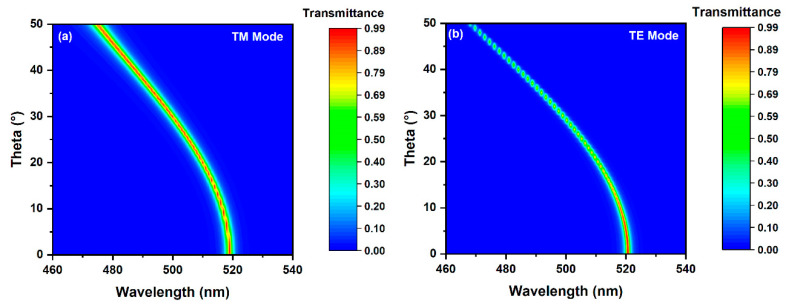
Evolution of the transmissive spectrum in TM (**a**) and TE (**b**) as the incident angle increases.

Figure 11 shows the evolution of the transmissive spectrum as the angle of incidence with different polarization modes increases from 0° to 50°. An evident shifting of the center wavelength occurs as the angle of incidence is greater than 10°. The variation almost reaches 50 nm for both of the modes, as shown in Figure 11, suggesting that the device is sensitive to the incident angle. Another important message from Figure 11 is that the evolution trends of the transmissive spectra in TM & TE modes are consistent. There is only a difference in the intensities of the transmissive spectra. The similar evolution trends in different modes are attributed to the isotropy of light response of the film in the x-y plane. 

The results of the simulation manifest that the filter based on the proposed structure in this paper presents an excellent performance. Moreover, Gudovskikh, A. S. et al. reported that GaP could be grown on a Si wafer by low-temperature plasma-enhanced atomic layer deposition [42]. As for ZnS and SiO_2_, all these materials can be deposited by atomic layer deposition according to other researchers’ reports [49,50]. Accordingly, it provides us a feasible way to fabricate the device in the same chamber at one time without breaking down the vacuum. As a result, the device investigated in this paper also has a potential value for a practical application in the future. 

## 4. Conclusions

In summary, a transmissive filter composed of GaP/SiO_2_ DBR and ZnS/SiO_2_ is simulated. An ultra-narrow linewidth of spectrum in the visible range is obtained according to the results of the simulation. The FWHMs of transmission peaks corresponding to the center wavelengths at 465 nm, 520 nm, and 620 nm reach 6.70 nm, 2.35 nm, and 6.91 nm, respectively. Because the entire device is designed based on dielectric materials, a very low total absorption in the visible range is achieved. The transmittance of the device can reach 0.75, 0.82, and 0.90 at 465 nm, 520 nm, and 620 nm. In comparison with traditional MDM structure, the narrower linewidth and higher transmittance result in more saturated primary colors. Finally, a larger scale of the color gamut can be realized in comparison with that of Adobe RGB color gamut. Additionally, the device also shows polarization-independent characteristics, which is very suitable for high-resolution imaging systems, detectors, and spectrometers.

## Figures and Tables

**Table 1:** The spectral data of the FP cavities with various thicknesses of SiO_2_ interlayer.

Thickness of SiO_2_ (nm)	Center Wavelength (nm)	Maximum Transmittance	FWHM (nm)
128	465	0.756	6.46
144	484	0.833	3.11
156	500	0.841	3.02
170	520	0.822	2.05
186	545	0.889	2.97
196	561	0.867	2.93
206	576	0.856	2.59
216	590	0.847	2.82
234	620	0.902	5.35
248	636	0.911	6.45
